# Development of a clinical calculator to aid the identification of MODY in pediatric patients at the time of diabetes diagnosis

**DOI:** 10.1038/s41598-024-60160-0

**Published:** 2024-05-08

**Authors:** Beverley M. Shields, Annelie Carlsson, Kashyap Patel, Julieanne Knupp, Akaal Kaur, Des Johnston, Kevin Colclough, Helena Elding Larsson, Gun Forsander, Ulf Samuelsson, Andrew Hattersley, Johnny Ludvigsson

**Affiliations:** 1 The Department of Clinical and Biomedical Sciences, Faculty of Health and Life Sciences, University of Exeter, Exeter, UK; 2 Skånes University Hospital, Lund, Sweden; 3 Faculty of Medicine, Imperial College London, London, UK; 4 Exeter Genomics Laboratory, The Royal Devon University Healthcare NHS Foundation Trust, Exeter, UK; 5 Department of Clinical Sciences Malmö, Lund University, Lund, Sweden; 6 Department of Pediatrics, Skånes University Hospital, Malmö, Sweden; 7 Department of Paediatrics, Institute for Clinical Sciences, Sahlgrenska Academy, University of Gothenburg, Gothenburg, Sweden; 8 Region Västra Götaland, Department of Paediatrics, Sahlgrenska University Hospital, Queen Silvia Children’s Hospital, Gothenburg, Sweden; 9 Crown Princess Victoria Children’s Hospital and Division of Pediatrics, Linköping University, Linköping, Sweden

**Keywords:** Genetics, Diseases, Endocrinology, Medical research

## Abstract

Maturity Onset Diabetes of the Young (MODY) is a young-onset, monogenic form of diabetes without needing insulin treatment. Diagnostic testing is expensive. To aid decisions on who to test, we aimed to develop a MODY probability calculator for paediatric cases at the time of diabetes diagnosis, when the existing “MODY calculator” cannot be used. Firth logistic regression models were developed on data from 3541 paediatric patients from the Swedish ‘Better Diabetes Diagnosis’ (BDD) population study (n = 46 (1.3%) MODY (*HNF1A, HNF4A, GCK*)). Model performance was compared to using islet autoantibody testing. HbA1c, parent with diabetes, and absence of polyuria were significant independent predictors of MODY. The model showed excellent discrimination (c-statistic = 0.963) and calibrated well (Brier score = 0.01). MODY probability > 1.3% (ie. above background prevalence) had similar performance to being negative for all 3 antibodies (positive predictive value (PPV) = 10% v 11% respectively i.e. ~ 1 in 10 positive test rate). Probability > 1.3% and negative for 3 islet autoantibodies narrowed down to 4% of the cohort, and detected 96% of MODY cases (PPV = 31%). This MODY calculator for paediatric patients at time of diabetes diagnosis will help target genetic testing to those most likely to benefit, to get the right diagnosis.

## Introduction

Maturity Onset Diabetes of the Young (MODY) represents between 1 and 3% of patients diagnosed with diabetes in the paediatric age range in large national and regional studies throughout the world^1–5^. MODY is diagnosed by genetic testing but this is expensive, prohibiting universal screening, so approaches are needed to identify those most likely to have it. Making a diagnosis of MODY in the childhood population is difficult as there are multiple clinical features that are suggestive but none are diagnostic on their own. This means hard specific binary criteria, as used in neonatal diabetes (i.e. test all patients diagnosed before 6 months^6^), are inappropriate and unworkable. One solution to this was to develop the “MODY calculator”, a probability model based solely on the specific values of clinical criteria (such as BMI, age of diagnosis, parental diabetes etc.) that would identify those at the greatest risk of MODY so they could be referred for diagnostic genetic testing^7^. The calculator was developed to be used in young-onset patients between the ages of 1 and 35^7^.

The MODY calculator is available online (https://www.diabetesgenes.org/exeter-diabetes-app/) and is widely used internationally and features in multiple national and international guidelines^8–12^. However, it was developed on cross-sectional data and can only be used more than 6 months after diagnosis as a key clinical feature it uses is whether the patient is insulin treated within 6 months^7^. Ideally, diagnosis of MODY would be made as close to the time of diabetes diagnosis as possible as it can completely alter a patient’s treatment and management. In children, who will have a lifetime ahead of them, it is crucial to not misdiagnose MODY as Type 1 diabetes.

Identifying the people who will ultimately be insulin dependent is difficult at diagnosis due to the “honeymoon” period, especially as the usual current policy is to give insulin even to patients with very low insulin requirement. At diagnosis, islet autoantibodies are highly discriminatory between MODY and Type 1 diabetes as MODY patients do not have Type 1 diabetes related autoantibodies^13^. This was not a feature of the original model, but would be particularly advantageous to include in a model for the paediatric population, where the majority of patients will be insulin treated and the prior probability of Type 1 diabetes is at its highest.

The Better Diabetes Diagnosis (BDD) study ^14^ provides an ideal population dataset in which to build a clinical prediction model for MODY at diagnosis, as it has recorded all the clinical features at diagnosis of diabetes and extensive molecular genetic testing for the three most common types of MODY in paediatric diabetes (the forms that allow a major change in treatment). We report the results of developing a new MODY calculator for use at diagnosis in the paediatric population using the BDD data set.

## Method

### Participants and data

We used data from the 3933 individuals diagnosed with diabetes between 1 and 18 years of age who were recruited as part of the Swedish national consecutive prospective cohort ‘Better Diabetes Diagnosis’ (BDD) study^14^, which involved 42 hospital paediatric clinics in Sweden and recruited from May 2005 to December 2010. All methods presented below were carried out in accordance with relevant guidelines and regulations.

### Routine clinical features (model predictors)

For the development of the prediction models we used clinical data collected at diagnosis (age at diagnosis, BMI, symptoms of polyuria, polydipsia, weight loss, diabetes ketoacidosis (DKA), parental history of diabetes and HbA1c) and islet autoantibody results (GADA, IA-2A, ZnT8A). Where data on symptoms at diagnosis were missing (n = 56), we assumed the patient had not experienced those symptoms. Statistical models were built using data from 3541 individuals who had complete data on key clinical features.

HbA1c was analysed locally with results returned within 24 h. Diabetic ketoacidosis (DKA) was defined as pH < 7.3 OR serum bicarbonate < 15mEq/L with a plasma glucose > 11mmol/L. Demographic data, symptoms, physical signs and blood analysis at onset were registered in SWEDIABKIDS, a national incidence and longitudinal quality control register for children and adolescents with diabetes^15^.

### Antibody testing (model predictors)

GADA, IA-2A, and ZnT8A were analyzed in radiobinding assays as described previously^1,16^. The cut-off values used equated to the level found in only 1% of an age-matched population. GADA and IA-2A levels were expressed as units per millilitre derived from the World Health Organization standard 97/550 and were considered positive if GADA levels were > 35 units/mL and IA-2A levels > 5 units/mL. The intra-assay coefficient of variation (CV) for duplicates was 5% for GADA and 11% for IA-2A. The radioligand binding assays for all three ZnT8A variants (ZnT8RA, ZnT8WA, and ZnT8QA) were analyzed. Cut-off values for positive results were ZnT8RA ≥ 75 units/mL, ZnT8WA ≥ 75 units/mL, and ZnT8QA ≥ 100 units/mL. The intra-assay CV was 5.5% for ZnT8RA, 5.3% for ZnT8WA, and 4.9% for ZnT8QA.

The laboratory undertaking the autoantibody analyses participates in the biannual Islet Autoantibody Standardization Program (http://www.immunologyofdiabetessociety.com/).

### Genetic testing for MODY (model outcome)

As described in the original study^1^, of the 3933 participants, 81 were selected for MODY testing on clinical grounds, and an additional 404 with sufficient DNA available (227 autoantibody negative, 177 autoantibody positive) were tested on research grounds, with those who received testing having similar clinical characteristics to those who did not receive testing^1^.

The coding exons and conserved splice sites of HNF1A, HNF4A, and GCK were amplified by PCR and sequenced on an ABI 3730 (Applied Biosystems, Warrington, U.K.). Sequences were compared with the published reference sequences (NM_000545.6 for HNF1A, NM_175914.4 for HNF4A, and NM_000162.5 for GCK) using Mutation Surveyor v3.24 (SoftGenetics, State College, PA) or ABI SeqScape Software v2.5 (Applied Biosystems). Variants were classified according to the American College of Medical Genetics and Genomics guidelines^17^. MODY was diagnosed by the identification of heterozygous pathogenic or likely pathogenic variants. A total of 46 cases tested positive for one of the three major MODY genes (29 *GCK*, 10 *HNF1A*, 7 *HNF4A*). This led to a prevalence of MODY of 1.3% in the 3541 with complete data for model building.

### Type 1 diabetes cohort for external model testing

We analysed participants with a clinician-assigned diagnosis of type 1 diabetes (age at diagnosis range 4–18 years) recruited as part of the UK-wide ADDRESS-2 study who had data available for the final model^18^. We only analysed participants who had an HbA1c result within 8 weeks of diagnosis. Participants whose HbA1c results were taken > 8 weeks after diagnosis were not included as the model is to be used at time of diagnosis and may be affected by treatment after this point. The detailed protocol for the study has been published previously^18^. DNA and serum samples were collected alongside clinical characteristics and symptoms at diagnosis. All antibody negative patients were tested for MODY genes using next generation sequencing^19^. External validation was limited due to the low number of MODY patients, but we used this cohort to explore the numbers that would be tested at different probability thresholds from the model and present the positive predictive value for MODY, with 95% confidence intervals.

### Statistics

#### Sample size calculation

We performed sample size calculations using the pmsampsize package in R, in line with the approach described by Riley et al.^20^. The original MODY models^7^ had a c-statistic on external validation of 0.94. For this model, for a c-statistic of 0.9, up to five model parameters, and a prevalence of 1.2%, a sample size of 1225 would be sufficient for estimation with a low margin of error (0.05) and targeted shrinkage of 0.9.

#### Statistical approaches for model development and performance

We developed multivariable prediction models to discriminate MODY from non-MODY patients, using complete-case analysis. Recognising the imbalance in the dataset due to the low prevalence of MODY, the prediction models were built using Firth Logistic Regression with Added Covariate (FLAC) ^21^, which is a more robust approach to address concerns of overfitting given the small number of MODY cases. Firth logistic regression implements a penalized maximum likelihood approach to reduce potential bias in the coefficients, but requires an added covariate recalibration step as the approach biases predicted probabilities towards 0.5. Predictors were added into the model using backward selection and the best model was determined using the Likelihood Ratio Test.

We assessed discrimination of the models through ROC curves. Due to the low prevalence of MODY in this cohort meaning the model probabilities would be very low, model calibration was carried out by splitting into groups (deciles apart from the top 20% where the MODY cases were which were split into further quintiles to provide greater granularity) and we compared the mean probability in each group against the proportion of MODY and presented 95% confidence intervals. We also present calibration statistics including Brier Score, calibration intercept and slope, and the Spiegelhalter Z-test.

We also present thresholds for number of MODY detected against proportion of the cohort tested in both the whole cohort, and just those who are antibody negative.

To determine potential clinical utility, we examined different thresholds of MODY probability that could be used for clinical decision making (1.3% (representing the prevalence of MODY in the cohort), and 5%, 10% and 20% as examples of what would happen if basing decisions on higher probability thresholds). For each threshold, we calculated the proportion of the cohort that would be tested if using that threshold to prioritise MODY testing (i.e. testing only those above that probability threshold), the proportion of MODY that would be detected, and the positive test rate. We examined the same statistics for if combining these thresholds with antibody testing.

#### Bootstrap internal validation

We took 1000 bootstrap samples with replacement to determine model optimism. We also examined how the thresholds of 1.3%, 5%, 10% and 20% would impact on the proportion of MODY detected/proportion of cohort tested for the different bootstrap samples and models.

#### Testing of model in ADDRESS-2

ADDRESS-2 provided an independent dataset of patients at diagnosis with MODY at population prevalence level. As we were only analysing those whose HbA1cs were within 8 weeks of diagnosis, the sample size was relatively small with only two patients with MODY. This meant validation was limited and we were therefore unable to provide validation of the discriminatory performance and calibration of the models, but it did offer an external dataset in which to examine the proportions that would be tested at different thresholds and to compare this to BDD.

#### Ethics

The BDD study was approved by the Ethics Committee at Karolinska Institutet (no 04-826/1 with amendments 2006/108-32/1 and 2007/1383-32/1, 2009/1684/32 and 2011/1069-32). Ethics approval for the ADDRESS-2 study was granted by the South Central—Berkshire NHS Research Ethics Committee on 3 October 2010 (ref: 10/H0505/85). Ethics approval for monogenic testing in the ADDRESS-2 cohort was granted by the East of England—Essex Research Ethics Committee on 14 July 2016 (ref. 16/EE/0306).

Informed consent was obtained from all subjects and/or their legal guardian(s).

## Results

### Clinical features for discriminating MODY from non-MODY

**Figure 1:**
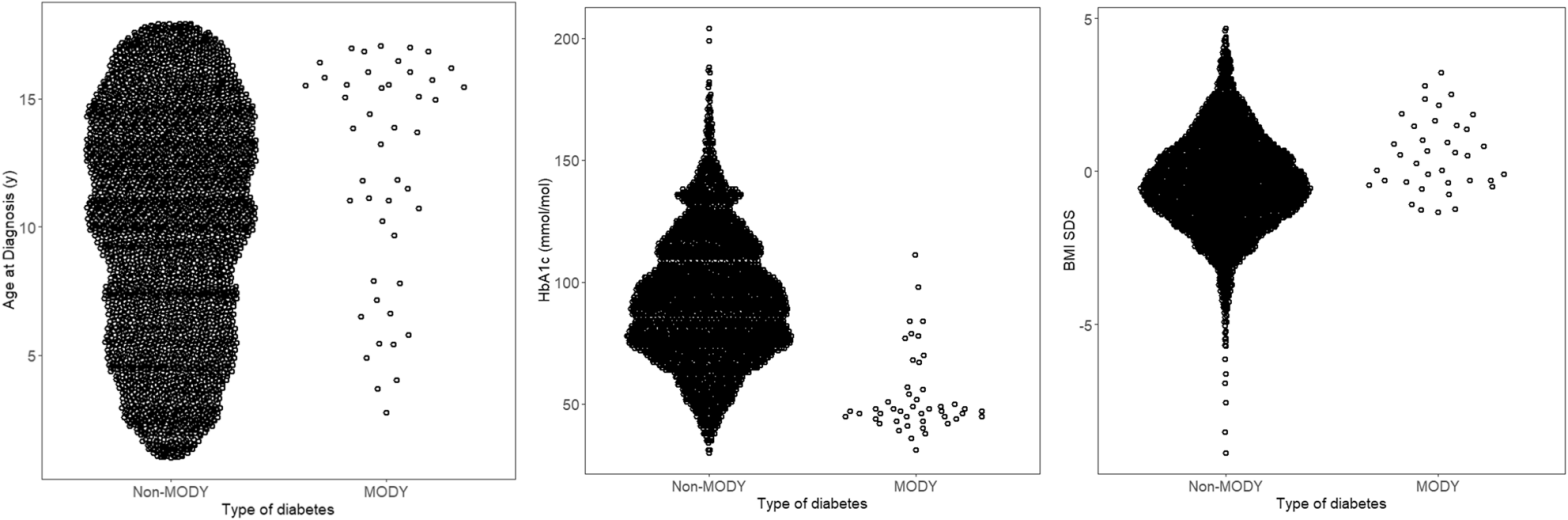
Beeswarm plots showing distributions of clinical features at diagnosis for MODY and non-MODY patients: (**a**) age at diagnosis, (**b**) HbA1c, and (**c**) BMI standard deviation score (SDS). For each plot, the points representing the MODY patients are shown on the right and the points representing the Non-MODY patients are shown on the left.

Figure 1 shows the continuous features in BDD and their distributions for MODY and non-MODY patients. Patients with MODY had similar ages at diagnosis and BMI as patients with type 1 diabetes, so were poor at discriminating between MODY and non-MODY patients (ROC AUC = 0.64 and 0.68, respectively). In addition, BMI was missing in 551 (14%) (including 8 MODY patients (17% of MODY)) therefore it was not included in any further analysis. Patients with MODY had a lower HbA1c than non-MODY patients showing good discriminatory value overall (ROC AUC = 0.91). HbA1c information was missing on 392 (10%) participants (age and sex was similar in those with and without missing data (10.1 v 10.1, *p* = 0.9 by t test, respectively) and sex (57.7% male v 55.1%, *p* = 0.4 by chi-squared test)). As this was a key differential factor between MODY and non-MODY patients initial prediction models based on clinical features were developed based on the cohort of 3541 patients with complete data (which included all 46 MODY cases), leading to a prevalence of MODY in this cohort of 1.3%.

Table 1a shows the binary clinical features in BDD and their sensitivity, specificity, positive and negative predictive values (PPV and NPV respectively) for discriminating MODY from non-MODY patients.

**Table 1:** Binary features at diagnosis and their predictive value for identifying MODY.

Criteria	Prevalence of criteria			Sensitivity (proportion with MODY meeting criteria)	Specificity (proportion without MODY not meeting criteria)	ROC AUC	PPV for MODY (proportion of people meeting criteria who have MODY)	NPV (proportion of people not meeting criteria who do not have MODY)
	All (n = 3541)	MODY (n = 46)	Non-MODY (n = 3495)					
(a) Clinical features								
HbA1c < 58	8%	78%	7%	78.3% [63.6, 89.1]	93.0% [92.1, 93.8]	0.856	12.8% [9.1, 17.3]	99.7% [99.4, 99.9]
Female Sex	45%	54%	45%	54.4% [39.0, 69.1]	55.3% [53.6, 56.9]	0.548	1.6% [1.0, 2.3]	98.9% [98.4, 99.3]
Parent affected	14%	63%	13%	63.0% [47.6, 76.8]	87.0% [85.8, 88.1]	0.75	6.0% [4.0, 8.5]	99.4% [99.1, 99.7]
Absence of Polyuria	7%	72%	6%	71.7% [56.5, 84.0]	93.8% [92.9, 94.5]	0.828	13.2% [9.2, 18.0]	99.6% [99.3, 99.8]
Absence of polydipsia	8%	72%	7%	71.7% [56.5, 84.0]	92.9% [92.0, 93.7]	0.823	11.7% [8.2, 16.1]	99.6% [99.3, 99.8]
Absence of DKA	84%	100%	83%	100% [92.3, 100]	16.7% [15.5, 18.0]	0.583	1.6% [1.1, 2.1]	100% [99.4, 100]
Absence of Weight loss	28%	87%	27%	87.0% [73.7, 95.1]	72.7% [71.2, 74.2]	0.80	4.0% [2.9, 5.4]	99.8% [99.5, 99.9]
(b) Islet autoantibodies								
GAD only tested Negative	46%	100%	45%	100% [92.3, 100]	54.8% [53.1, 56.5]	0.774	2.8% [2.1, 3.8]	100% [99.8, 100]
IA2 only tested Negative	30%	100%	29%	100% [92.3, 100]	70.9% [69.4, 72.4]	0.855	4.3% [3.2, 5.7]	100 [99.9, 100]
ZnT8 only tested Negative	39%	100%	38%	100% [92.3, 100]	62.2% [60.6, 63.8]	0.811	3.4% [2.5, 4.5]	100 [99.8, 100]
All 3 antibodies negative	12%	100%	11%	100% [92.3, 100]	89.4% [88.4, 90.4]	0.947	11.1% [8.2, 14.5]	100 [99.9, 100]

PPV = positive predictive value, NPV = negative predictive value. All features are at time of diagnosis.

Absence of DKA had the highest sensitivity in MODY patients (as none of the MODY patients had DKA), but it had poor positive predictive value as only 1.6% of those without DKA had MODY. Absence of polyuria and absence of polydipsia had much higher positive predictive values for MODY whilst still maintaining high specificity and negative predictive values. Parent affected was a weaker predictor with a positive predictive value for MODY of 6.0%. For comparison with previous work^1^, we show that an HbA1c < 58 mmol/mol at diagnosis has 78% sensitivity (95% CI 64, 89%) for detecting MODY with a positive predictive value of 12.8%. However, this can be used continuously in a probability model to improve prediction.

### Antibodies for discriminating MODY from non-MODY

Antibody negativity had high sensitivity for MODY, but in isolation the positive predictive value was relatively weak (2–5%), indicating a single negative antibody has low probability for MODY (Table 1b). The positive predictive value improved further in those who were negative for all 3 antibodies (11% of patients negative for all 3 antibodies had MODY).

### Prediction models

In FLAC logistic regression models, only parent affected by diabetes, HbA1c and absence of polyuria were independently associated with log odds of MODY (see Supplementary Table S1 for final model coefficients). Despite its high sensitivity, absence of DKA did not add to the models (β (95% CI) = 1.1 (− 1.05, 5.92), *p* = 0.75), likely relating to the poor positive predictive value (PPV) for MODY and its association with HbA1c which was already captured in the model.

The model showed excellent discrimination (c-statistic = 0.963 (95% CI 0.951, 0.976), Supplementary Table S2 and Supplementary Figure S1). The model also showed good calibration with predicted probabilities lining up well with observed numbers of MODY (Supplementary Table S3), a low Brier Score, and good fit with calibration intercept and slope close to 0 and 1, respectively (Supplementary Table S2). MODY probabilities were higher in the 29 GCK-MODY patients compared to the 17 patients with mutations in the *HNF1A* or *HNF4A* gene (mean probability 22% v 10%, respectively, *p* = 0.02).

### Comparison of approaches for identifying MODY patients

If all patients who were negative for all 3 autoantibodies were selected for genetic testing (n = 416) this would find all MODY cases (n = 46) and approximately 1 in 11 patients tested would be positive.

In Table 2 we show the proportion of MODY detected and proportion of cohort tested at different thresholds of MODY probability from the model. If testing all individuals at a probability > 1.3% (i.e. probability above background prevalence of MODY), you would test 12% of the cohort (i.e. a similar proportion to testing all antibody negative), and would pick up 93% of MODY cases. Using higher thresholds of 5%, 10% or 20% would involve testing progressively fewer people and a higher positive test rate for MODY, but would result in more cases of MODY being missed. In line with the mean probabilities, using higher thresholds for testing was more likely to pick up GCK-MODY, with the majority of HNF1A/HNF4A-MODY cases only being detected when using the lower thresholds (using 20% threshold, 2/17 HNF1A/4A MODY cases picked up compared with 13/16 GCK-MODY cases; using 1.3% threshold, 15/17 HNF1A/4A-MODY cases picked up compared with all 29 GCK-MODY cases).

**Table 2:** Comparison of approaches for identifying MODY patients.

	Proportion MODY detected	Proportion of cohort tested	Positive test rate for MODY
MODY Prob > 1.3%	44/46 96%	433/3541 12%	44/433 10%
MODY Prob > 5%	35/46 76%	209/3541 6%	35/209 17%
MODY Prob > 10%	23/46 50%	105/3541 3%	23/105 22%
MODY Prob > 20%	15/46 33%	53/3541 1.5%	15/53 28%
Negative all 3 antibodies	46/46 100%	416/3541 11.7%	46/416 11.1%

### Combining the prediction model and antibody testing:

The MODY prediction model could be used to screen patients to select subgroups of the population for further antibody testing. Table 3 shows the impact of using different probability thresholds with antibody testing to narrow down the cohort for genetic testing. Within the cohort, 433 (12%) had a MODY probability > 1.3% and of these 140 were found to be negative for all three antibodies (4% of the whole population). This would mean only 4% of the population would need testing if only referring those with a probability > 1.3% who were antibody negative. There was a 31% positive test rate for MODY in that group. Using higher probability thresholds from the model would mean fewer individuals would need antibody testing and genetic testing, but this would result in more MODY cases being missed.

**Table 3:** At different probability thresholds for MODY, the proportions of population negative for all 3 antibodies, and the population tested and positive test rate for MODY if testing all above that probability threshold.

	Proportion above that probability who would be negative for all 3 antibodies	Proportion of whole cohort who are above that probability threshold AND negative for all 3 antibodies	Positive test rate for MODY if testing all above that threshold AND negative for all 3 antibodies	Proportion MODY detected
MODY Prob > 1.3%	140/433 32%	140/3541 4.0%	44/140 31%	44/46 96%
MODY Prob > 5%	97/209 46%	97/3541 2.7%	35/97 36%	35/46 76%
MODY Prob > 10%	53/105 50%	53/3541 1.5%	23/53 43%	23/46 50%
MODY Prob > 20%	31/53 58%	31/3541 0.9%	15/31 48%	15/46 33%

If performing antibodies on all patients 416/3541 (12%) were negative for all three antibodies. In this subgroup, the mean MODY probability from the prediction model was 4.8%.

If using the > 1.3% MODY probability thresholds, 140 cases would be tested (32% of the antibody negative, 4% of the original cohort) and 44 (96%) of MODY cases would be picked up.

#### Internal validation

In internal validation, the model was found to have low levels of optimism (ROC curve optimism = 0.003). Based on three different approaches for bootstrap internal validation, the proportion MODY detected and proportion of cohort tested estimates were relatively stable (Supplementary Table S4).

#### External validation in ADDRESS-2

When applying the model in the ADDRESS-2 cohort, there were 205 participants under the age of 18 with data available for the model who had HbA1c results taken within eight weeks of diagnosis. Two of these patients had MODY (1% prevalence). This cohort had similar characteristics to the BDD cohort (see Supplementary Table S5), with the main difference relating to reported polyuria which in ADDRESS-2 was reported as polyuria/polydipsia. The model probabilities for the two MODY patients were 3.4% and 5.0%. Of this cohort, 19/205 (9.3% [95% CI 5.7%, 14.1%]) had a MODY probability > 1.3%, similar to the proportion seen in BDD (12%). Furthermore, using this threshold led to a similar positive predictive value (2/19 (10.5%) participants above > 1.3% probability had MODY in ADDRESS-2 compared with 10% in BDD). Of the 19 patients with MODY probability > 1.3%, 6 (32%) were negative for all 3 antibodies, including the two MODY cases (so a pick up rate of 2/6 (33%) if using both antibodies and the probability model to select patients for testing). Only four patients (2%) had a probability > 5% and this would pick up one of the MODY patients (as the probability was 5.0002%).

#### Online calculator

The model equations have been turned into an online calculator available at https://julieanneknupp.shinyapps.io/mody_bdd/. The online calculator allows the user to add the clinical features of an individual (parent affected with diabetes, HbA1c at the time of diagnosis and presence of polyuria at the time of diagnosis) and will return the probability of MODY based on these features using the equations from the statistical models presented in this paper. The user can also add in optional antibody test results, if they are available that will alter the probabilities accordingly. Model coefficients for all equations for the online calculator are shown in Supplementary Table S6.

## Conclusion/Discussion

We have shown how using a probability model at diagnosis combining simple clinical features of HbA1c, parent affected, and polyuria can be used to help identify those most likely to have MODY to prioritise testing. We illustrate how by using different thresholds from the model, along with antibody testing, you can reduce down to a group of patients where positive test rates for MODY are around 1 in 3, and very few MODY cases are missed.

This study had key strengths. The analysis was performed on a population based dataset with > 90% recruitment rate. This means we had large numbers available and the probabilities obtained from the model will be appropriate for use in a population setting. Crucially, unlike the original MODY calculator, this version can be used from the time of diagnosis and the online calculator includes antibody results, to help ensure MODY patients can get the right diagnosis for their diabetes as early as possible and reduce the burden of unnecessary insulin treatment. In addition, the real advantage of the model is that it allows combinations of clinical features and weights them appropriately to return probabilities of MODY, which can be particularly helpful in cases where the phenotype is less clear e.g. some MODY cases have higher HbA1cs which will reduce the probability, but if this is accompanied by a parent with diabetes and absence of polyuria, the probability will increase accordingly.

There were a number of limitations. Although the sample size was large, due to the rarity of MODY, the proportion of MODY cases was small and external validation was on limited numbers. However, the sample size was still deemed appropriate for obtaining estimates with a low margin of error, and we used a robust regression modelling approach to allay concerns of overfitting. We used internal validation to obtain estimates of uncertainty in our outcomes. We were limited in external validation due to the lack of datasets available with measures taken at the time of diabetes diagnosis and population testing of MODY. This meant full validation of the discrimination and calibration of the models was not possible. In the validation dataset there were no cases under the age of 4, and, in contrast to the development dataset, HbA1c was tested up to 8 weeks after diagnosis. However, the mean and standard deviation of the baseline HbA1c was similar in the two cohorts so this was unlikely to have a major effect on our results (Table S5). There were only 2 MODY cases in the external validation dataset, however, this reflects the population prevalence level of MODY and therefore allowed us a limited assessment of how the model may work in practice in terms of proportions who would be put forward for genetic testing based on different probability thresholds.

In addition, the low prevalence of MODY means that the probabilities from the model are low on average, but this reflects population prevalence of the disease and we illustrate how, together with antibody testing, even with these low model probabilities you can use the models to narrow down to a cohort where around 1 in 3 of those tested would have MODY.

This model is only suited for use at diagnosis and therefore relies on documentation of features at diagnosis, particularly HbA1c and polyuria. It is important that later HbA1cs are not used as the performance is likely to be weaker then, as treatment and the honeymoon period lower the HbA1c in Type 1 diabetes, reducing its discriminatory power.

This model was developed on a mainly white European population. Although the prevalence of MODY was low in this population, it is similar to that seen in other paediatric cohort studies^2–4^ indicating the probabilities are likely to be appropriate on average. However, the applicability may be different in populations with higher rates of DKA or young-onset type 2 diabetes, where the clinical picture is likely to be very different. Further validation in these populations would be needed to determine whether recalibration or model updating is required.

With increasing knowledge of type 1 diabetes in the community and with possible future screening programs, diagnosis of diabetes can be made in an earlier stage of disease. In previous studies it has been shown that children followed before diagnosis often are non-symptomatic and have low HbA1c, while autoantibodies are positive^22,23^. Since these children could be considered as having MODY if they have a parent with diabetes, it will be important to always take autoantibodies into account.

Finally, the model was only developed on a dataset with the common forms of MODY (HNF1A, GCK and HNF4A) and not rarer causes. However, the common forms of MODY are the ones that affect treatment so it is these cases that are the most important to diagnose. Also as they are the most common, they are more cost-effective to test for.

Better approaches to identifying MODY at diagnosis have been needed. Previous work has indicated a strong family history and very mild diabetes could lead to suspicions in rare cases, but these criteria could miss cases so a probability based approach has advantages to help prioritise patients for expensive genetic tests. The model we have developed shows good discrimination for MODY compared with non-MODY at the time of diabetes diagnosis, with a probability > 1.3% (above background prevalence level) having a similar positive predictive value for MODY as being negative for GAD, IA2 and ZnT8 islet antibodies. Based on our data provided, a policy could be developed for clinical care balancing the resources available for testing against proportion of MODY cases detected. Higher probabilities can be used to narrow down the proportion tested and a higher pick up rate for MODY in those tested, but at the expense of missing more MODY cases in the proportion not tested. We have also shown how this model can be used in combination with islet-autoantibody testing to further improve the positive test rate, whilst limiting the number of MODY cases missed. There is clear evidence that even a single positive islet autoantibody makes MODY highly unlikely^13^. Testing of 3 islet autoantibodies in all paediatric patients at diagnosis should be considered, if funding permits. If the funding system (state supported or insurance based) cannot afford to test all antibody negative subjects then our model provides an excellent way to maximise the yield from limited DNA testing based on routinely available information.

The probability model for MODY at diagnosis of diabetes that we have developed uses key features not used in the previous MODY calculator including HbA1c at diagnosis and markers of symptoms, which relate to the severity of hyperglycaemia at diagnosis in Type 1 diabetes compared with MODY. These features are likely to be particularly discriminatory in patients with mutations in the *GCK* gene where glucose homeostasis is still maintained but at a slightly higher set-point^24^. These features are likely to be less informative once treatment is initiated after diagnosis, and particularly as patients with Type 1 diabetes enter the honeymoon period. Therefore, it is critical for this model that the HbA1c used is the one taken at time of diagnosis. Clinically patients with the common forms of MODY do not tend to present with DKA^25^ and MODY can therefore be excluded. However DKA did not add much in terms of predictive power in our dataset, most likely because of its low positive predictive value association with high HbA1c and polyuria.

There are clear advantages to getting the diagnosis of MODY right from the start. Treatment of Type 1 diabetes is a heavy burden for the patients and in children for their families. Insulin is given, being lifesaving in Type 1 diabetes, but with several serious adverse events and risks^26^. Modern devices such as smart pumps and glucose sensors have made the treatment more efficacious, but these are both expensive and very demanding. In contrast, patients with *HNF1A-*MODY or HNF4A-MODY are very sensitive to sulphonylurea tablets^27^, and patients with GCK-MODY do not require any pharmacological treatment at all^28^. It is therefore extremely important to avoid patients with MODY being incorrectly diagnosed as having Type 1 diabetes and therefore receiving unnecessary insulin treatment. MODY is hard to diagnose with 77% of cases estimated to be misdiagnosed many years after diagnosis^29^. Even though the proportion of MODY among newly diagnosed children with diabetes is low (~ 1%)^1^, this means hundreds of new cases every year, and identifying them from diagnosis is vital to ensure they are treated optimally from the outset.

In conclusion, we have developed a probability model to help identify paediatric patients most likely to have MODY that can be used at the time of diagnosis. This model can be used in isolation, or in combination with antibody testing, to prioritise genetic testing.

### Supplementary Information

Supplementary Information.

## Data Availability

BDD data involved in this analyses are available after reasonable request and ethical approval from the senior author (JL) upon approval of the BDD steering group. ADDRESS-2 data access is available via a management committee^18^.
