# Supplementary material for: Development of a clinical calculator to aid the identification of MODY in pediatric patients at the time of diabetes diagnosis
Source: Sci Rep. 2024 May 8;14:10589. doi: 10.1038/s41598-024-60160-0 (PMC11079008; doi:10.1038/s41598-024-60160-0)
Supplement: Supplementary file 1 — Supplementary Information. [file 41598_2024_60160_MOESM1_ESM.docx]

**Online only supplement to accompany Shields et al. “Developing a “MODY calculator” for use in the paediatric population at the time of diabetes diagnosis**

**eMethods 1 – Further details on islet autoantibody testing**

GADA and IA-2A levels were expressed as units per millilitre derived from the World Health Organization standard 97/550 and were considered positive if GADA levels were >35 units/mL and IA-2A levels >5 units/mL. The intra-assay coefficient of variation (CV) for duplicates was 5% for GADA and 11% for IA-2A. The radioligand binding assays for all three ZnT8A variants (ZnT8RA, ZnT8WA, and ZnT8QA) were analyzed. Cut-off values for positive results were ZnT8RA ≥75 units/mL, ZnT8WA ≥75 units/mL, and ZnT8QA ≥100 units/mL. The intra-assay CV was 5.5% for ZnT8RA, 5.3% for ZnT8WA, and 4.9% for ZnT8QA.

The laboratory undertaking the autoantibody analyses participates in the biannual Islet Autoantibody Standardization Program (<http://www.immunologyofdiabetessociety.com/>).

**eMethods 2:**

Recognising the imbalance in the dataset due to the low prevalence of MODY , the prediction models were built using Firth Logistic Regression with Added Covariate (FLAC) (20), which is a more robust approach to address concerns of overfitting given the small number of MODY cases. Firth logistic regression implements a penalized maximum likelihood approach to reduce potential bias in the coefficients, but requires an added covariate recalibration step as the approach biases predicted probabilities towards 0.5.

We assessed discrimination of the models through ROC curves. Due to the low prevalence of MODY in this cohort meaning the model probabilities would be very low, model calibration was carried out by splitting into groups (deciles apart from the top 20% where the MODY cases were which were split into further quintiles to provide greater granularity) and we compared the mean probability in each group against the proportion of MODY and presented 95% confidence intervals. We also present calibration statistics including Brier Score, calibration intercept and slope, and the Spiegelhalter Z-test.

We also present thresholds for number of MODY detected against proportion of the cohort tested in both the whole cohort, and just those who are antibody negative.

To determine potential clinical utility, we examined different thresholds of MODY probability that could be used for clinical decision making (1.3% (representing the prevalence of MODY in the cohort), and 5%, 10% and 20% as examples of what would happen if basing decisions on higher probability thresholds). For each threshold, we calculated the proportion of the cohort that would be tested if using that threshold to prioritise MODY testing (i.e. testing only those above that probability threshold), the proportion of MODY that would be detected, and the pick-up rate. We examined the same statistics for if combining these thresholds with antibody testing.

**Table S1 -** Regression coefficients for the final prediction model. Beta coefficients represent the log odds ratio per unit increase in the predictor and are the components of the regression equation used for the model predictions. Odds ratio is the exponential of the beta coefficients and represents the increase in odds of MODY per unit increase in the predictor (with binary variables coded as 1 and 0).

|  | β (95% CI) | Odds Ratio (95% CI) | Chi-sq | p |
| --- | --- | --- | --- | --- |
| Intercept | -1.42 (-3.08, 0.24) | 0.24 (0.04, 1.27) | 2.8 | 0.09 |
| Parent with diabetes | 1.63 (0.99, 2.29 S) | 5.11 (2.69, 9.90) | 24.8 | 6 x 10^-8^ |
| HbA1c (mmol/mol) | -0.06 (-0.08, -0.04) | 0.94 (0.92, 0.96) | 35.2 | 3 x 10^-10^ |
| Absence of polyuria | 1.91 (1.16, 2.70) | 6.75 (3.18, 14.93) | 25.8 | 4 x 10^-8^ |

Figure S1– Beeswarm plot showing the probabilities of MODY from the final regression model for MODY and non-MODY patients.


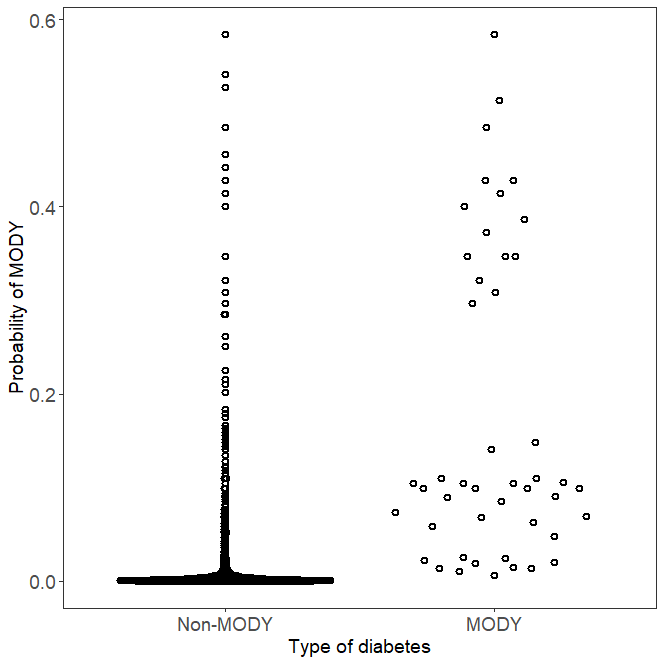


Table S2 - Model performance statistics:

| c-statistic | 0.963 |
| --- | --- |
| Brier Score | 0.011 |
| R^2^ | 0.412 |
| Calibration intercept | 0.051 |
| Calibration slope | 1.02 |
| Somers’ Dxy | 0.927 |
| Spiegelhalter z test | z=0.45, p=0.65 |

Table S3 - Calibration of MODY model – Data presented in subgroups defined based on deciles, with the top 20% split further by quintiles to give more detailed breakdown in the ranges where there are MODY cases.

| MODY probability group | Number of individuals in that group | Mean probability within that decile | N (%) MODY cases [95% CI] |
| --- | --- | --- | --- |
| 0.0002% - 0.018% | 371 | 0.0093% | 0 |
| 0.018% - 0.037% | 338 | 0.027% | 0 |
| 0.037% - 0.067% | 358 | 0.050% | 0 |
| 0.067% - 0.11% | 369 | 0.086% | 0 |
| 0.11% - 0.17% | 399 | 0.14% | 0 |
| 0.17% - 0.26% | 329 | 0.21% | 0 |
| 0.26% - 0.35% | 328 | 0.30% | 0 |
| 0.35% - 0.59% | 355 | 0.46% | 0 |
| 0.59% - 0.88% | 157 | 0.7% | 1 (0.64%) [0.016, 3.5] |
| 0.88% - 1.4% | 121 | 1.1% | 2 (1.7%) [0.2, 5.8] |
| 1.4% - 2.9% | 139 | 2.0% | 7 (5.0%) [2.0, 10.1] |
| 2.9% - 8.1% | 141 | 5.2% | 6 (4.3%) [1.6, 9.0] |
| 8.1% - 58.4% | 136 | 21.3% | 30 (22.1%) [15.4, 30.0] |

Table S4 - Results of internal validation:

Proportion of MODY patients detected and proportion of cohort tested using three different probability thresholds for the original dataset and model, and 95% range of estimates based on internal validation using 1000 bootstrap samples: Case 1 = probabilities based on predictions from the bootstrap model applied to the bootstrap sample; Case 2 = probabilities based on predictions from original model on the bootstrap sample; Case 3 = probabilities based on bootstrap model applied to original sample

|  | **Proportion MODY detected** | | | | **Proportion of cohort tested** | | | |
| --- | --- | --- | --- | --- | --- | --- | --- | --- |
|  | **Original** | **Case 1** | **Case 2** | **Case 3** | **Original** | **Case 1** | **Case 2** | **Case 3** |
| MODY Prob >1.3% | 96% | 84.4%, 100% | 89.1%, 100% | 82.6%, 97.8% | 12% | 7.9%, 15.4% | 11.2%, 13.3% | 8.0%, 15.1% |
| MODY Prob >5% | 76% | 59.5%, 86.7% | 63.6%, 88.4% | 63.0%, 78.3% | 6% | 4.3%, 7.2% | 5.2%, 6.7% | 4.4%, 6.9% |
| MODY Prob >10% | 50% | 28.6%, 76.6% | 36.2%, 64.8% | 32.6%, 69.6% | 3% | 1.9%, 4.8% | 2.4%, 3.6% | 2.0%, 4.6% |
| MODY Prob >20% | 33% | 18.9%, 48.8% | 19.6%, 46.5% | 30.4%, 39.1% | 1.5% | 1.0%, 2.2% | 1.1%, 1.9% | 1.0%, 2.1% |

Table S5 – Baseline characteristics of ADDRESS2 participants compared to BDD participants

|  | BDD (n=3541) | ADDRESS-2 (n=205) |
| --- | --- | --- |
| Age at diagnosis (y)  Mean (SD) | 10.1 (4.4) | 12.3 (3.4) |
| Female % | 45% | 44% |
| Parent affected % | 14% | 14% |
| HbA1c^a^  Mean (SD) | 92.8 (26.2) | 89.3 (27.9) |
| Polyuria^b^ % | 7.1% | 1.5% |

^a^In ADDRESS-2 HbA1c was taken within 8 weeks of diagnosis, whereas the HbA1c was taken at the time of diagnosis in BDD. ^b^Polyuria in ADDRESS-2 was polyuria/polydipsia, whereas these were reported separately in BDD.

Table S6 – Model coefficients for all regression equations for the online calculator.

| Model | Intercept | Parent with diabetes (1=Y, 0=N) | HbA1c (mmol/mol) | Polyuria (1=Y, 0=N) | GAD result (1=Positive, 0=Negative) | IA2 result (1=Positive, 0=Negative) | ZnT8 result (1=Positive, 0=Negative) |
| --- | --- | --- | --- | --- | --- | --- | --- |
| Clinical Features only | 0.48606 | 1.63098 | -0.05731 | -1.90938 |  |  |  |
| Clin Feat + GAD | 1.13947 | 1.56918 | -0.05633 | -1.93789 | -4.67469 |  |  |
| Clin Feat + IA2 | 0.94204 | 1.33703 | -0.05028 | -1.62049 |  | -4.74651 |  |
| Clin Feat + ZnT8 | 0.67422 | 1.51953 | -0.05014 | -1.70407 |  |  | -4.40205 |
| Clin Feat + GAD + IA2 | 1.02677 | 1.35035 | -0.04478 | -1.56825 | -4.20242 | -4.27647 |  |
| Clin Feat + GAD + ZnT8 | 0.92226 | 1.49842 | -0.04645 | -1.73035 | -4.31897 |  | -3.88539 |
| Clin Feat + IA2 + ZnT8 | 0.87521 | 1.23331 | -0.04605 | -1.43451 |  | -3.84262 | -3.07285 |
| Clin Feat + GAD, IA2 & ZnT8 | 0.92464 | 1.23597 | -0.04144 | -1.42063 | -4.03121 | -3.38322 | -2.22650 |
